# Preparation of superhydrophobic sponge and its application in oil-water separation and treatment of polymer-containing wastewater

**DOI:** 10.3389/fbioe.2023.1234939

**Published:** 2023-07-26

**Authors:** Liuya Fang, Hui Xu, Zhihai Fan, Xiaopeng Duan, Qing Wang, Xiaoliang Bai, Jiao Peng, Shiwei Jin

**Affiliations:** ^1^ National Petroleum Tubular Goods Quality Supervision and Inspection Center, Key Laboratory of Petroleum Tubular Goods and Equipment Quality Safety for State Market Regulation, CNPC Tubular Goods Research Institute, Xi’an, Shaanxi, China; ^2^ Changqing Oilfield Branch No. 6 Gas Production Plant, Xi’an, Shaanxi, China; ^3^ Changqing Oilfield Branch No. 1 Oil Production Plant, Xi’an, Shaanxi, China; ^4^ Key Laboratory of Catalysis and Energy Materials Chemistry of Education, Hubei Key Laboratory of Catalysis and Materials Science, South-Central Minzu University, Wuhan, China

**Keywords:** super hydrophobic, water purification, wastewater treatment, oil—water separation, antibacterial

## Abstract

At present, the application prospect of superhydrophobic materials in oil-water separation, an-tibacterial and other aspects have attracted more and more attention. However, preparing a simple and low-cost superhydrophobic material remains a challenge. Using acetone as solvent, candle soot, silver/silica nanoparticles and polydimethylsiloxane were uniformly mixed to form a mixed solution, and the superhydrophobic sponge was successfully prepared by spraying method. The results show that the superhydrophobic sponge has high water contact Angle (162°) and excellent oil-water separation efficiency, which can realize effective treatment of polymerized wastewater. In addition, the superhydrophobic sponge showed better antibacterial properties on the surface of *Escherichia coli* and *Staphylococcus aureus*. In this work, a simple way to prepare superhydro-phobic oil-water separation material is proposed. The preparation process is green, the material is easy to obtain, and it is expected to be widely used in practical production.

## Graphical Abstract

**Figure:**
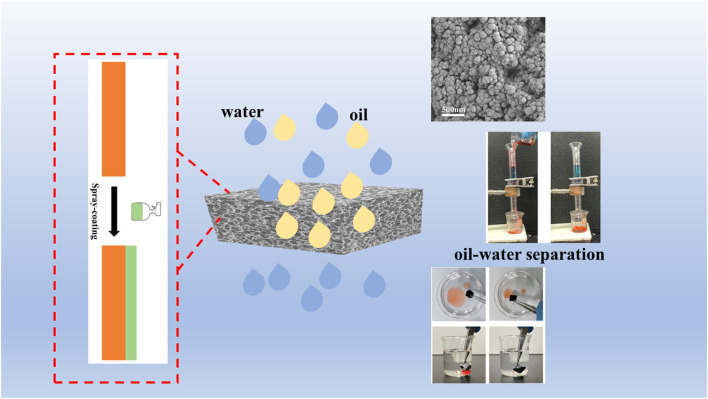

## 1 Introduction

With the rapid development of industrialization and the improvement of oilfield production technology, oil-bearing wastewater and organic wastewater have been continuously discharged into water bodies, causing serious environmental pollution and resource waste (Cao et al., 2019; Baig et al., 2022a; Kong et al., 2022). Among them, compared with conventional wastewater, polymer-containing wastewater in oilfield has complex composition and high emulsification stability (Liu, 2021; Qiao et al., 2022; Yu et al., 2022). The existing treatment technology is very limited, which makes the separation effect of oil and water unsatisfactory (Li et al., 2021). Therefore, researchers urgently need effective methods to solve such problems.

Superhydrophobic materials have become a research hotspot in the application of oil-water separation due to their special wettability (Ahuja et al., 2021; Farrokhi et al., 2021; Wang et al., 2021). Porous materials (fabrics, membranes, wood and sponges) have become ideal substrates for the preparation of ultra-wetting materials due to their wide sources, low cost and high adsorption capacity (Baig et al., 2022b; Kang et al., 2022; Pang et al., 2022; Wang et al., 2022). Yang et al. prepared a durable super hydrophobic cotton-based carbon fiber fabric. The combination of rough surface structure and low surface energy enables the cotton fabric to have super hydrophobic/super oil philic and self-cleaning properties. Various oil-water mixtures and emulsions can be separated under the action of gravity (Yang et al., 2022). Li et al. prepared hydrophobic mercaptan fossil mercaptan superhydrophobic sponge for oil-water separation by a simple dip drying process. Superhydrophobic sponges exhibit excellent absorption capacity up to 90 times their own weight (Zhang et al., 2017). Barthwal et al. proposed a simple method for preparing superhydrophobic/superoleophilic sponges modified with a zinc-based metal-organic skeleton (MOF-5) with oil-water separation and antibacterial properties. Superhydrophilic MOF-5 nanoparticles were hydrophobic by using low surface energy 1H, 1H, 2H, 2H- perfluorooctane trichlorosilane materials. The synergistic effect of H-MOF-5 and low surface PDMS resulted in superhydrophobicity. The sponges showed excellent oil-water separation and antibacterial properties. The superhydrophobic sponge can realize the separation of oil and water in two ways (adsorption and gravity driven separation) and the separation efficiency is >98% (Barthwal et al., 2022).

In this study, CS, Ag/SiO_2_ NPs and PDMS were used to prepare a green, durable and low-cost super hydrophobic sponge. Among them, CS provides hydrophobicity, synthetic Ag/SiO_2_ NPs adds antibacterial properties to the sponge, and PDMS serve as a bonding layer to connect CS, Ag/SiO_2_ NPs and the sponge. For different oil/organic solvo-water mixtures, the superhydrophobic sponge showed excellent oil/water separation efficiency (>99.5%) and good reusability and showed better treatment effect on oilfield polymer-containing wastewater. In addition, the super hydrophobic CS@Ag/SiO_2_ NPs@PDMS sponge also showed excellent antibacterial properties against *Escherichia coli* and *Staphylococcus aureus*, making it an ideal material for oil-water separation and antibacterial.

## 2 Materials and methods

### 2.1 Materials

Main reagents: candle (Wuhan Shentry Chemical Instrument Network Co., LTD.); Acetone (Analytically pure, Sinopharm Chemical Reagent Co., LTD.); Polydimethylsilox-ane (Sinopharm Chemical Reagent Co., LTD.); Tetraethyl orthosilicate (Sinopharm Chem-ical Reagent Co., LTD.); Ammonia (NH4OH, 28%, Sinopharm Chemical Reagent Co., LTD.); Silver nitrate (analytical pure, Sinopharm Chemical Reagent Co., LTD.); Sodium borohydride (Sinopharm Chemical Reagent Co., LTD.); Sudan Ⅲ (Sinopharm Group Chemical Reagent Co., LTD.); Polyaluminum chloride (Analytically pure, Sinopharm Chemical Reagent Co., LTD.); Ethanol, carbon tetrachloride, dichloromethane, cyclohex-ane, petroleum ether and n-hexane used in the experiment were all analytically pure and purchased from Sinopharm Chemical Reagent Co., LTD. Deionized water.

Main instruments: Contact Angle spectrometer (JC 2000D1), fourier transform infra-red spectrometer, FTIR Spectrometer (NEXUS-470), field emission scanning electron mi-croscope (FESEM) (HITACHI SU8010), ultraviolet spectrophotometer (A380), digital mag-netic heating agitators (ZNCL-BS), Ultrasonic cleaner (KQ5200E), electronic analytical balance (BSA224S-CW), tabletop high speed centrifuge (H1850), X-ray diffractometer.

### 2.2 Experimental method

#### 2.2.1 Experimental method of SiO_2_ NPs

SiO_2_ NPs was prepared by StÖber method (Xu et al., 2021). The mixture changed from clear to milky white turbid, indicating the formation of SiO_2_ colloidal particles. After constant temperature stirring reaction for 24 h, the colloidal solution was centrifuged, washed with distilled water and anhydrous ethanol five times successively, and finally dried in a vacuum drying oven at 50°C for 8 h to obtain SiO_2_ NPs.

#### 2.2.2 Experimental methods for Ag/SiO_2_ NPs

0.2 g SiO_2_ NPs were weighed and dissolved in 10 mL distilled water (250 mL conical flask) by ultrasonic dispersion, while 0.1 g silver nitrate was weighed in 10 mL distilled water and 0.01 g sodium borohydride was weighed in 100 mL distilled water, respectively, and stirred to fully dissolve them. After 10 min, the silver nitrate solution was added to the conical flask and placed in the magnetic agitator at a constant speed for 20 min at 40°C. The sodium borohydride solution was then added to the conical flask by drops and con-tinued to stir for half an hour after the drops were finished. Remove and cool to room temperature, wash with distilled water and anhydrous ethanol for five times successively, and finally dry in a vacuum drying oven at 50°C for 6 h to obtain Ag/SiO_2_ NPs.

#### 2.2.3 Preparation of superhydrophobic sponges

Candle soot (CS) is obtained by burning candles. Using acetone as solvent, CS, Ag/SiO_2_ NPs and PDMS were evenly mixed to obtain the mixed solution, and then the CS@Ag/SiO_2_ NPs@PDMs mixed solution was sprayed on the surface of sponge. Superhy-drophobic CS@Ag/SiO_2_ NPs@PDMS sponge was successfully prepared by drying it in the oven at 50°C.

#### 2.2.4 Adsorption capacity and oil-water separation performance

The adsorption properties of superhydrophobic sponges were evaluated by adsorp-tion experiments on various oils. The super hydrophobic CS@Ag/SiO_2_ NPs@PDMS sponge was placed in oil for 2 min to achieve adsorption saturation. The mass of superhydropho-bic CS@Ag/SiO_2_ NPs@PDMS sponge before and after adsorption was weighed. Absorption capacity Q_m_ can be calculated by the following formula:

$$Q_{m}=\frac{{m_{2-}m}_{1}}{m_{1}}$$ (1)

Q_m_ is adsorption capacity, m_1_ and m_2_ are respectively before and after adsorption for adsorption of super hydrophobic CS@Ag/SiO_2_ NPs@PDMS the quality of the sponge.

To evaluate the oil-water separation performance of the prepared superhydrophobic CS@Ag/SiO_2_ NPs@PDMS sponge, 10 mL of oil (dichloroethane and carbon tetrachloride) was mixed with 10 mL of water, and the oil-water mixture was poured into a homemade oil-water separation unit. Record the weight of water before and after separation, and cal-culate the separation efficiency according to Eq. 2

$$\eta =\frac{m_{1}}{m_{0}}\times 100\%$$ (2)

η (%) represents the separation efficiency of immiscible oil-water mixture, and m_0_ and m_1_ are the weight of water before and after separation, respectively.

Treatment of oilfield wastewater containing poly: 100 mL of sewage sample was mixed with appropriate amount of poly aluminum chloride and superhydrophobic sponge and stirred at 200 r/min for 5 min. Let stand for 1 h, then remove sponge and drain. Take appropriate amount of liquid for determination.

#### 2.2.5 Test for antibacterial properties

The experiment of the preparation of the bacteriostatic circle method is adopted to evaluate the CS@SiO_2_/Ag NPs@PDMS of gram-negative bacteria (*e. coli*) and gram-positive bacteria (*staphylococcus aureus*) antibacterial properties, choose LB agar solid culture medium. Under sterile conditions, an oxford cup was placed on the surface of a solid medium and 100 μL samples of antimicrobial agent were added to the oxford cup. The results were observed after 24 h of culture in a 37°C biochemical chamber, and the antibacterial zone was measured with a ruler.

## 3 Results and discussion

### 3.1 Morphology and chemical composition of SiO_2_ NPs and Ag/SiO_2_ NPs

**FIGURE 1:**
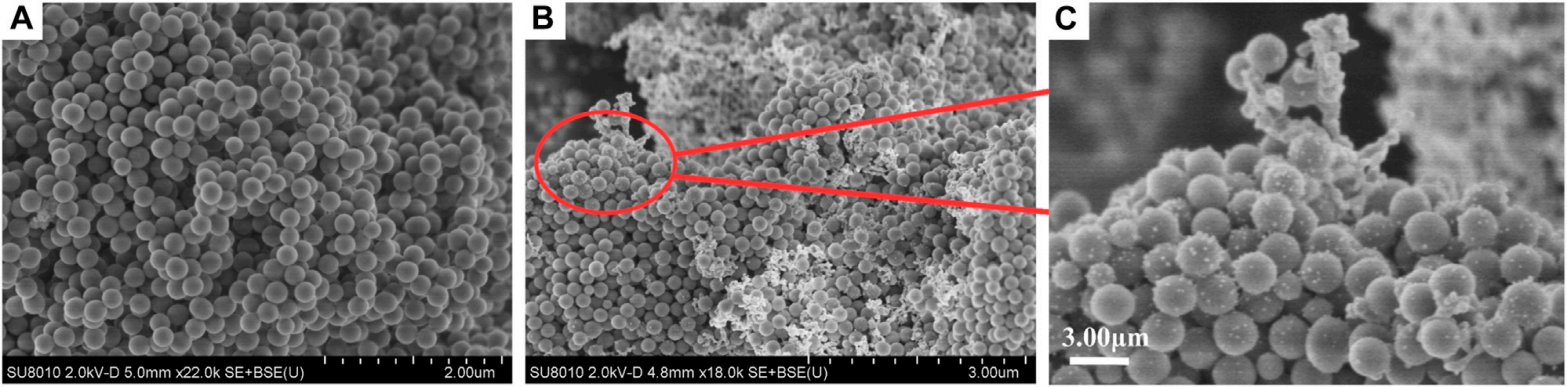
**(A)** SEM image of SiO_2_ NPs. **(B)** and **(C)** are SEM images of Ag/SiO_2_ NPs.Porous Materials.

The morphologies of SiO_2_ NPs and Ag/SiO_2_ NPs were characterized by SEM. As shown in Figure 1A, the prepared SiO_2_ NPs have smooth surfaces and are all spherical particles with a diameter of about 100–200 nm. Ag^+^ was reduced to the SiO_2_ NPs surface using sodium borohydride. Figures 1B, C show the morphology of Ag/SiO_2_ NPs. Ag NPs are uniformly distributed or clustered on the surface of SiO_2_ NPs.

**FIGURE 2:**
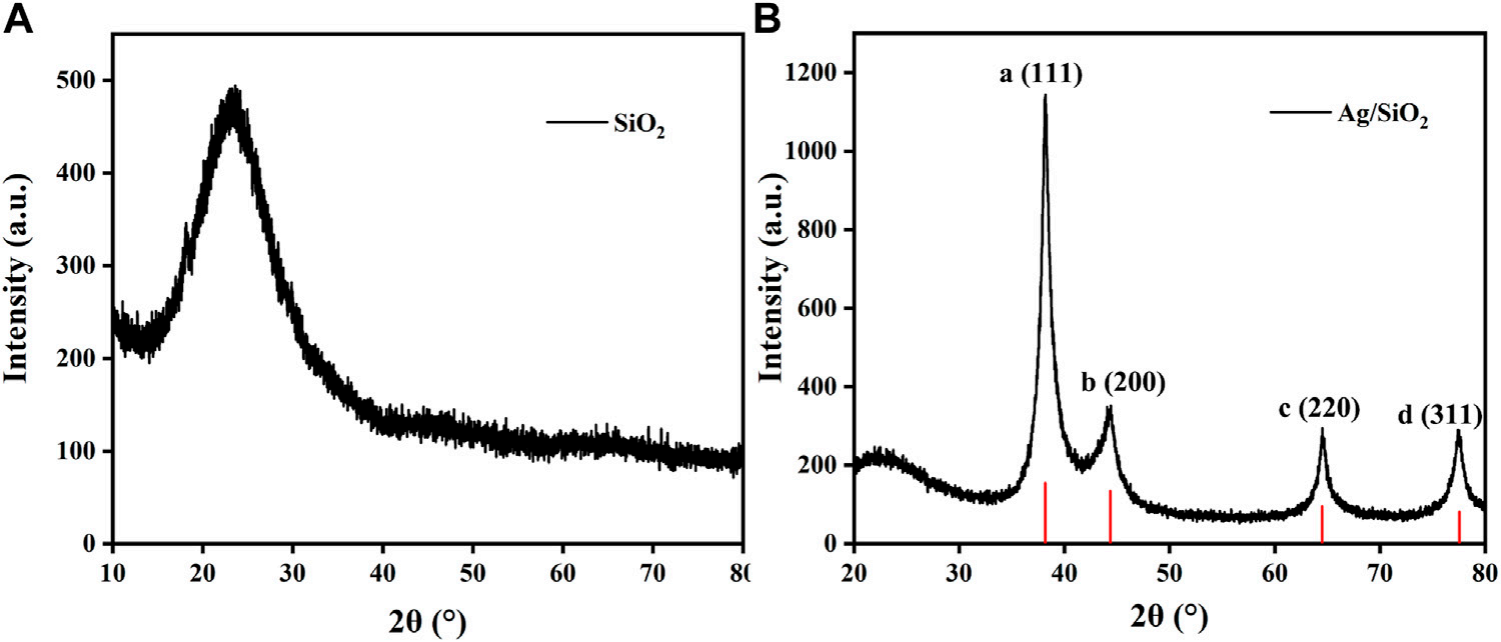
**(A)** XRD pattern of SiO_2_ NPs. **(B)** XRD pattern of Ag/SiO_2_ NPs.

Through SiO_2_ NPs samples of preparation of X ray diffraction analysis (XRD), all the diffraction peaks of amorphous samples are in the 20°–30° diffraction package (Figure 2A. As shown in Figure 2B, the positions of the five diffraction peaks of Ag/SiO_2_ NPs composites correspond to the (111), (200), (220) and (311) crystal planes of standard silver, respectively, which proves that Ag NPs in the synthesized Ag/SiO_2_ NPs composites has a good face-centered cubic structure.

**FIGURE 3:**
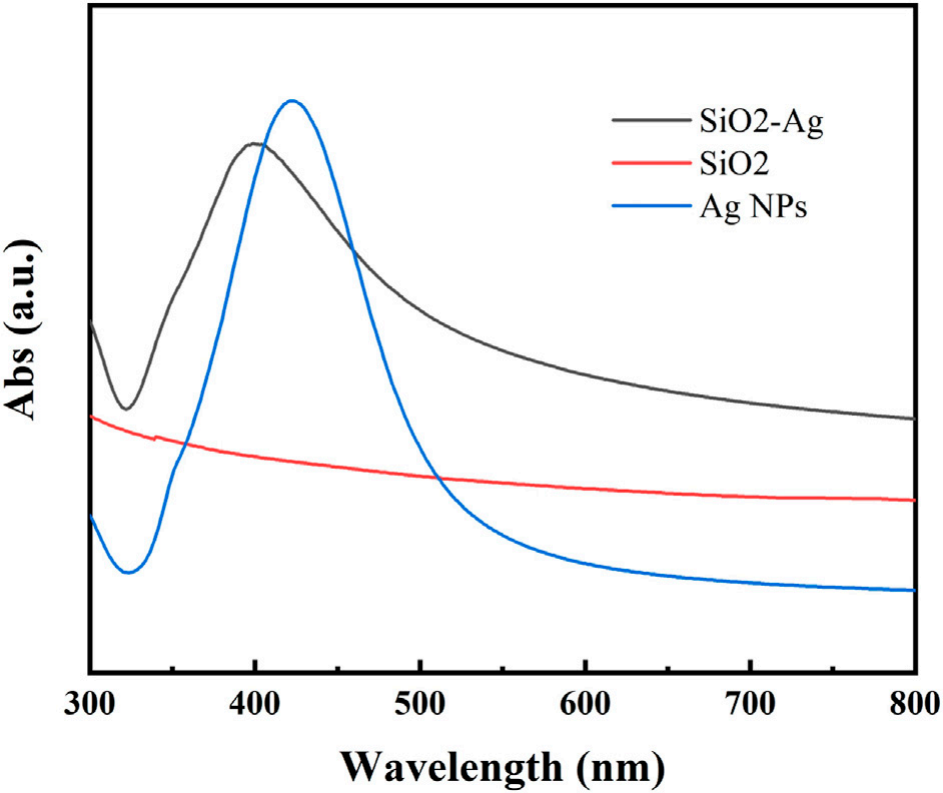
The FTIR diagram of Ag NPs, SiO_2_ NPs, and Ag/SiO_2_ NPs.

The prepared samples were analyzed by UV spectrophotometer (Figure 3). SiO_2_ NPs showed no obvious absorption peak within the test range, while Ag NPs had an absorption peak at 423.2 nm. The prepared Ag/SiO_2_ NPs showed a wide absorption peak near 403 nm, but no other absorption peaks, indicating that only Ag/SiO_2_ NPs were contained in the sample without Ag NPs.

### 3.2 Preparation of superhydrophobic CS@Ag/SiO_2_ NPs@PDMS sponge

#### 3.2.1 Preparation method of superhydrophobic sponge

**FIGURE 4:**
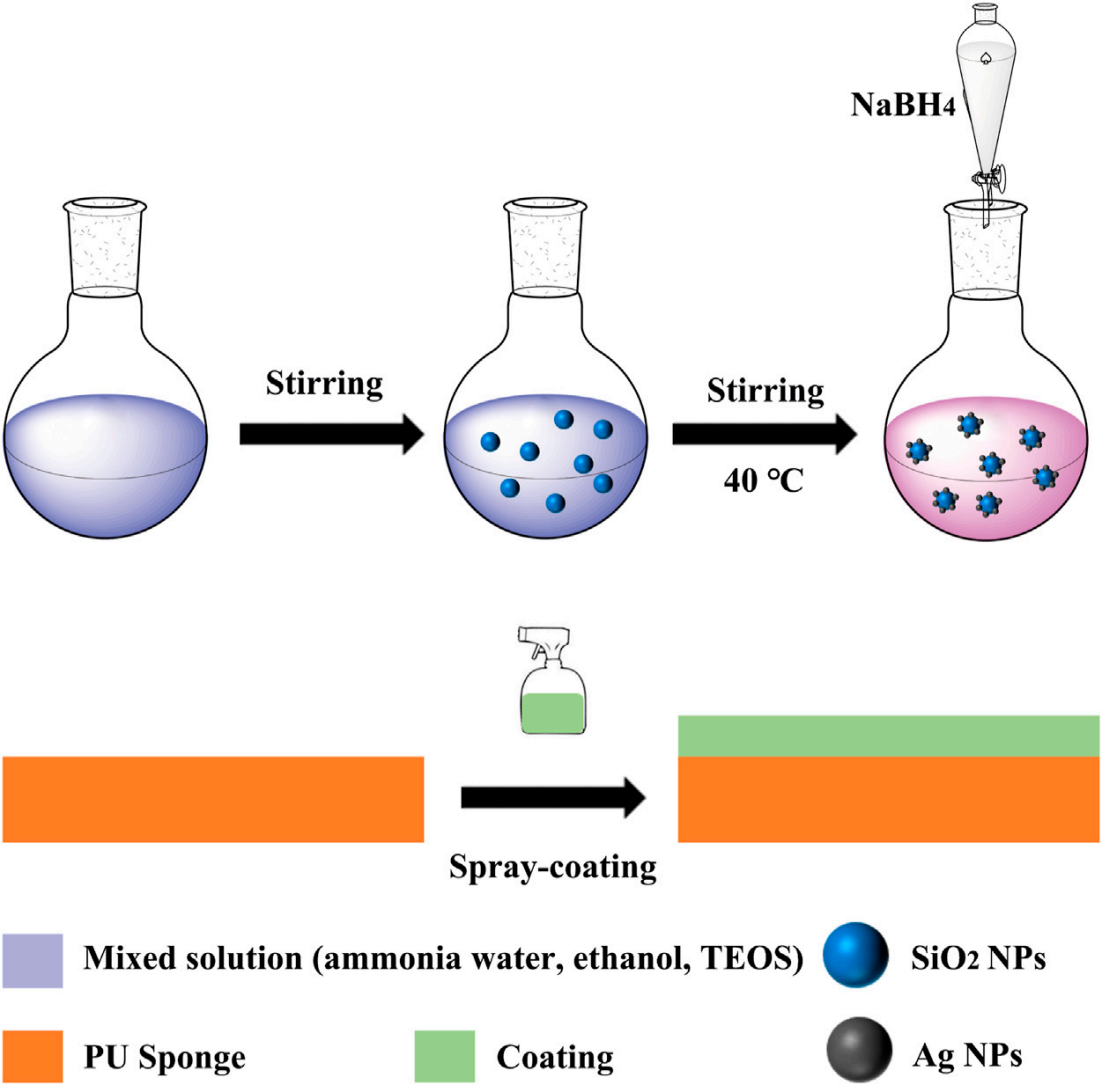
Preparation process of superhydrophobic surface.

Firstly, 4 g of CS and 0.2 g of Ag/SiO_2_ NPs were weighed and dispersed in 10 mL ace-tone solution, while 1 g of PDMS were weighed and dispersed in 5 mL acetone solution and stirred at 200 rpm for 10 min. Then mix the above two colloidal solutions and continue to stir for half an hour to get superhydrophobic paint. Commercial PU sponges were cut into the size of 1 × 1 × 1 cm^3^, ultrasonic cleaned 3 times in water-ethanol solution, and dried at 50°C for reserve. The sponge was sprayed with the superhydrophobic coating (5 mL) prepared above. The sponge was placed in an oven at 50°C and removed after 10 min. The spray-drying process was repeated three times. After drying to obtain a super hydro-phobic sponge. The whole preparation process is shown in Figure 4, which also includes the preparation process of SiO_2_ NPs and Ag/SiO_2_ NPs.

#### 3.2.2 Morphology and composition characterization of superhydrophobic sponges

**FIGURE 5:**
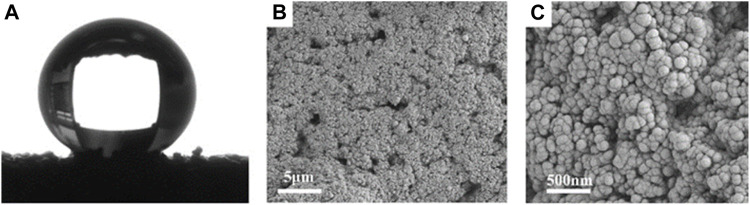
**(A)** The WCA of the superhydrophobic sponge. **(B)** SEM image of superhydrophobic sponge surface. **(C)** Enlarged SEM image of superhydrophobic sponge.

**FIGURE 6:**
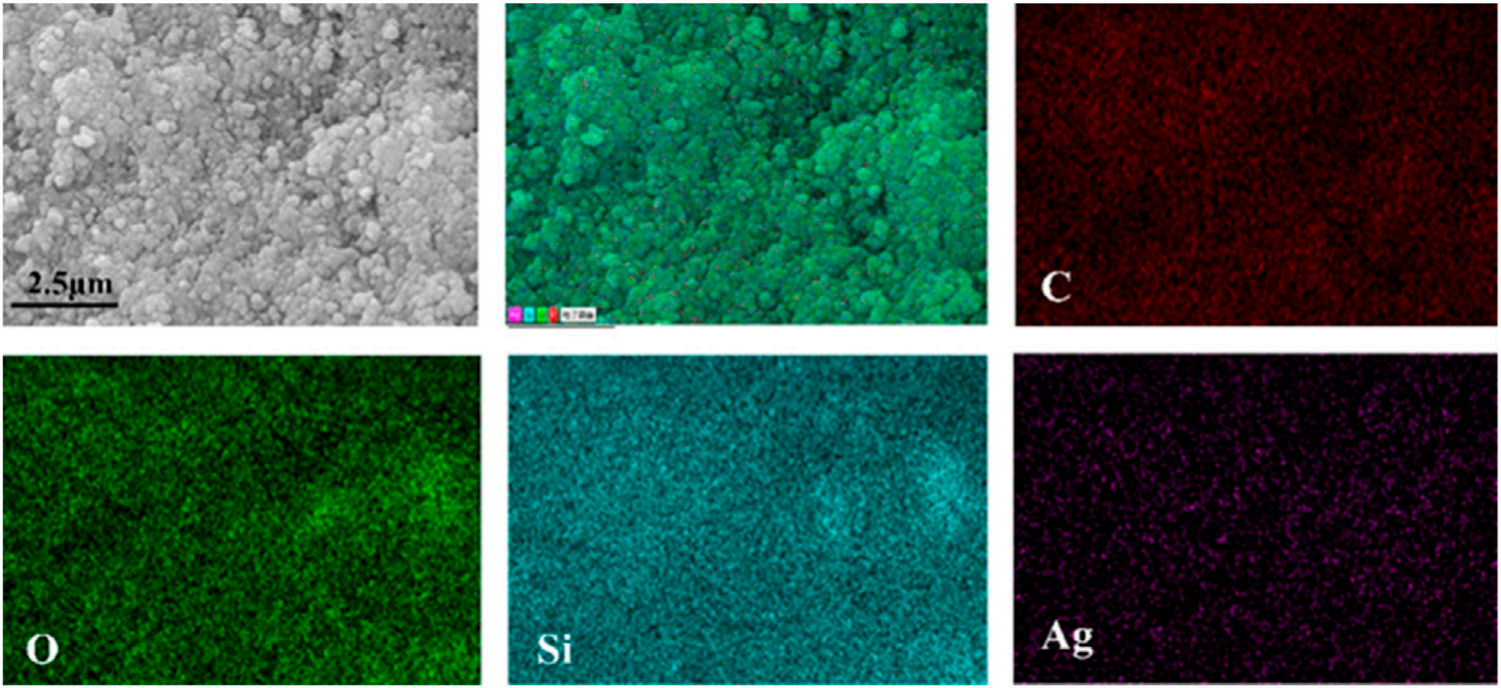
EDS maps of superhydrophobic sponge with C, O, Si and Ag uniformly distributed on the surface of the sponge.

**FIGURE 7:**
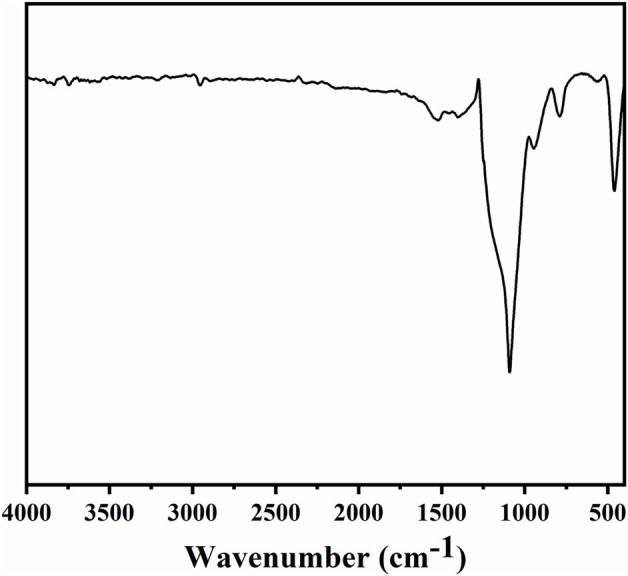
FTIR spectra of superhydrophobic sponge.

The morphology and chemical composition of coating surface are the key factors affecting wettability. The sponge surface was analyzed by SEM and EDS. As shown in Figure 5A, the WCA of the superhydrophobic CS@Ag/SiO_2_ NPs@PDMS sponge is about 162°. It was observed by SEM that the surface of the sponge was uniformly distributed with massive rough structures, and CS and Ag/SiO_2_ NPs were alternately distributed or clustered on the surface of the sponge (Figures 5B, C). As can be seen from the EDS image in Figure 6, C, O, Si and Ag are evenly distributed on the surface of the sponge. The chemical composition of the prepared samples was analyzed using FTIR (Figure 7). The strong absorption band at 1,094 cm^−1^ is attributed to the anti-symmetric stretching vibration of Si-O-Si, the bending vibration at 456 cm^−1^ corresponds to the bending vibration of Si-O in SiO_2,_ and the peak value at 786 cm^−1^ corresponds to the stretching and bending vibration of Si-C in PDMS.

**FIGURE 8:**
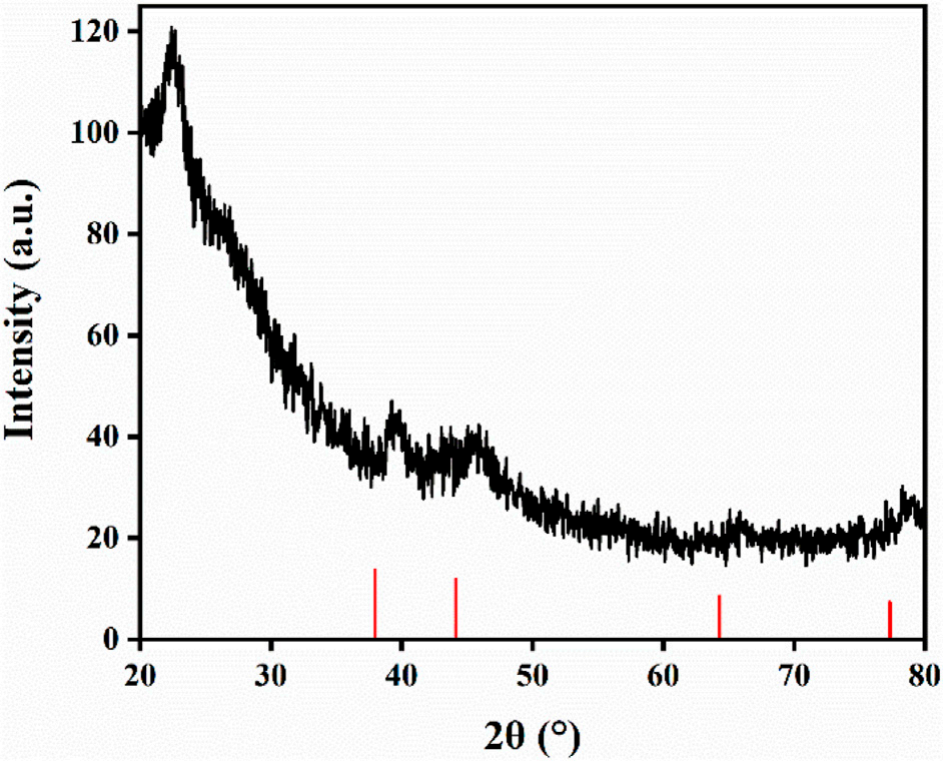
XRD spectra of superhydrophobic sponge.

Figure 8 shows the XRD characterization of the sponge sample. There are wide diffraction peaks near 2θ = 22.5°, which correspond to amorphous SiO_2_ and CS, and silver characteristic peaks appear at 2θ = 38.8°, 44.6°, 65.1° and 77.3°, indicating that CS and Ag/SiO_2_ NPs are successfully loaded on the surface of the superhydrophobic sponge. In conclusion, the superhydrophobic sponge was successfully prepared.

### 3.3 Oil-water separation performance test

**FIGURE 9:**
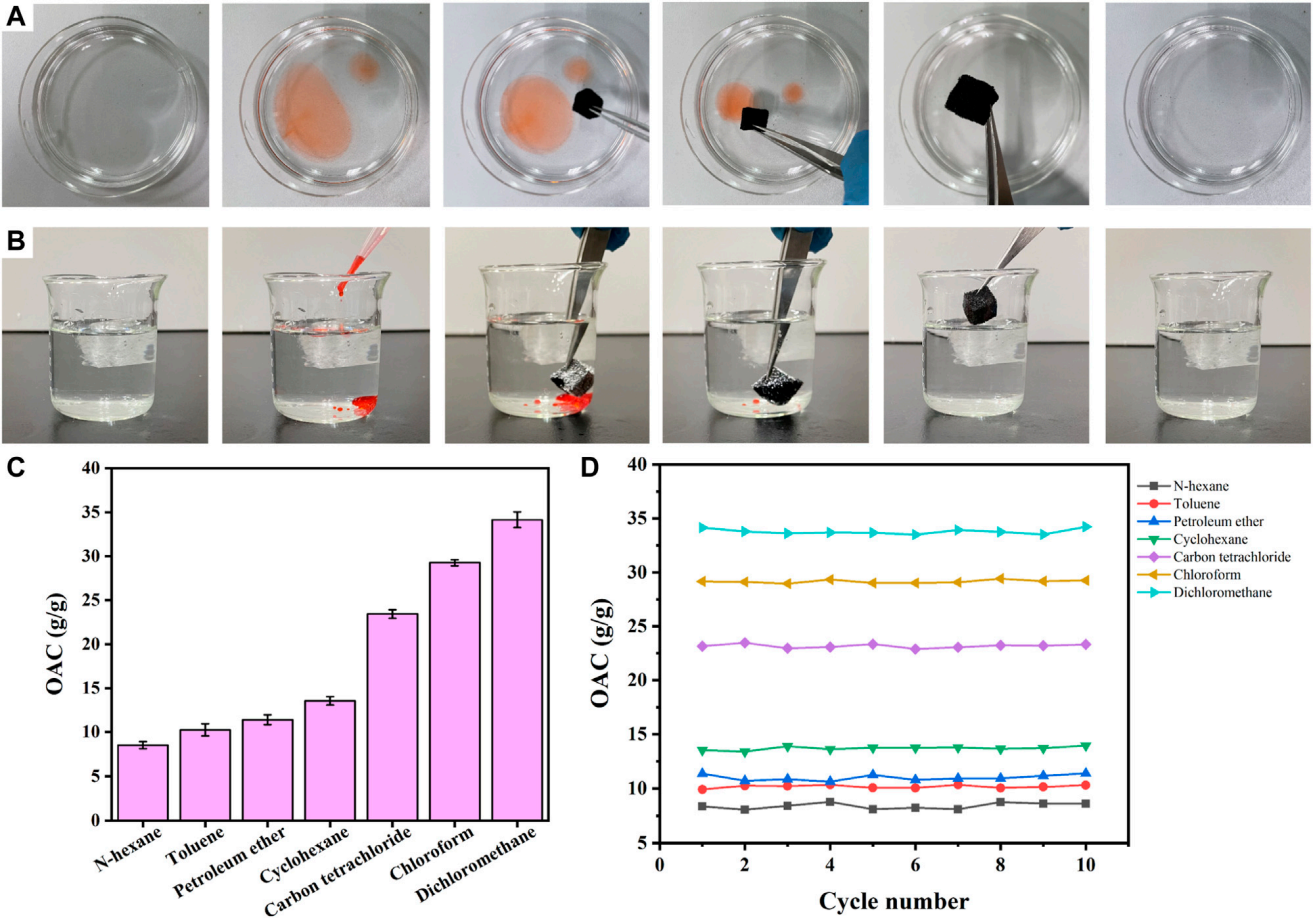
**(A)** The process of removing n-hexane from water by a superhydrophobic sponge. **(B)** Process diagram of carbon tetrachloride removal from water by a superhydrophobic sponge. **(C)** The absorptive capacity of different types of oils. **(D)** The absorption capacity of different types of oil under 10 cycles.

Ag/SiO_2_@PDMS@CS modified sponges have super hydrophobic and super oil philic properties with high absorption capacity and can selectively adsorb organic pollutants in water. The oil absorption capacity of superhydrophobic sponges was evaluated using light oil (n-hexane) and heavy oil (carbon tetrachloride). Figure 9A shows n-hexane floating on the water (stained with Sudan Ⅲ) being rapidly absorbed by a superhydrophobic sponge, leaving only clean water in the petri dish. Figure 9B shows that carbon tetrachloride (stained with Sudan Ⅲ) on the bottom is rapidly absorbed by a superhydrophobic sponge, leaving only clean water in the beaker. As shown in Figure 9C, different kinds of oils such as carbon tetrachloride, methylene chloride, cyclohexane, petroleum ether and n-hexane were also selected to test the oil absorption capacity of superhydrophobic sponge. Ag/SiO_2_@PDMS@CS modified sponge has the lowest absorption capacity of n-hexane, up to 8.4 times of its own mass. In contrast, the adsorption capacity of di-chloromethane was the highest, up to 34.5 times, which fully reflects the excellent oil absorption capacity of Ag/SiO_2_@PDMS@CS modified sponge. Ag/SiO_2_@PDMS@CS modified sponge has excellent recyclability, with its absorption capacity of various oils basically unchanged after 10 absorption cycles (Figure 9D).

**FIGURE 10:**
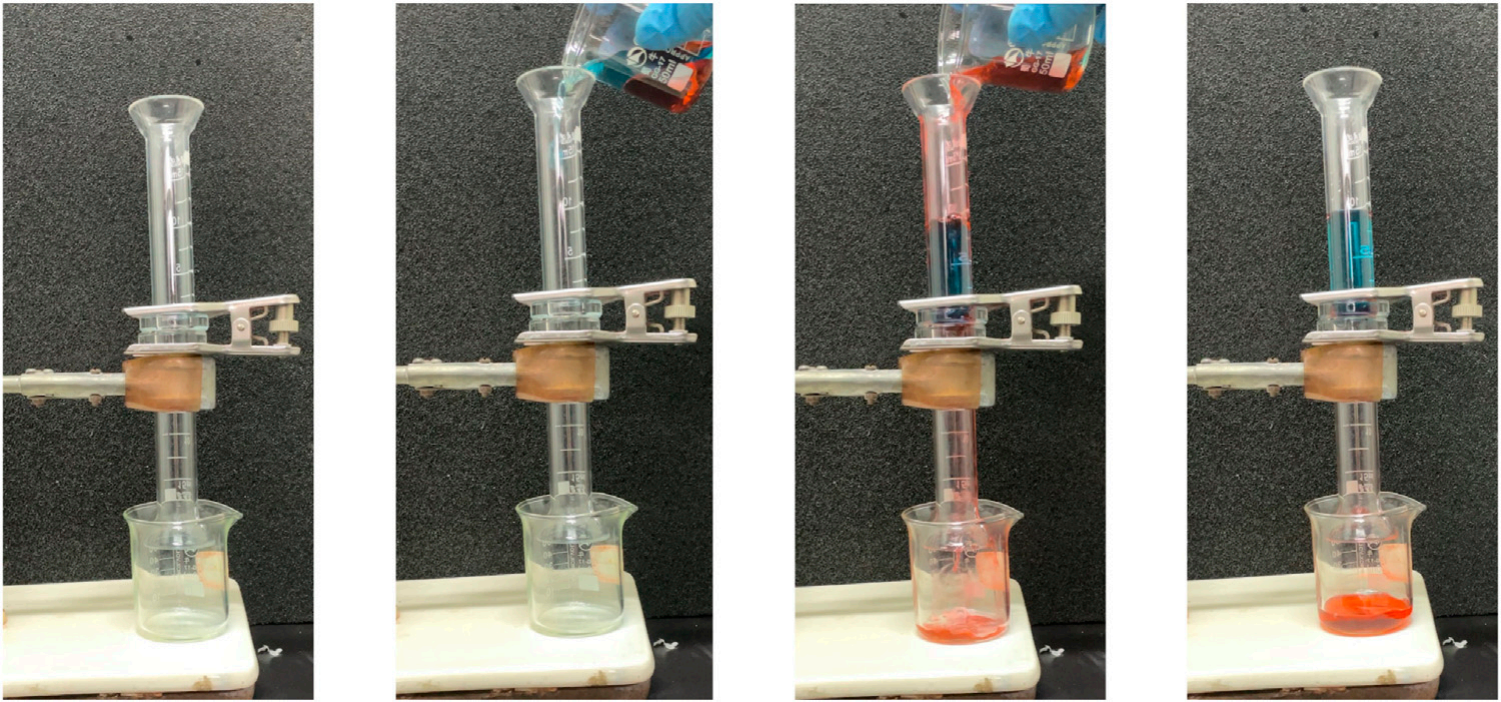
Diagram of the process of separating methylene chloride/water mixtures with superhydrophobic sponge.

**FIGURE 11:**
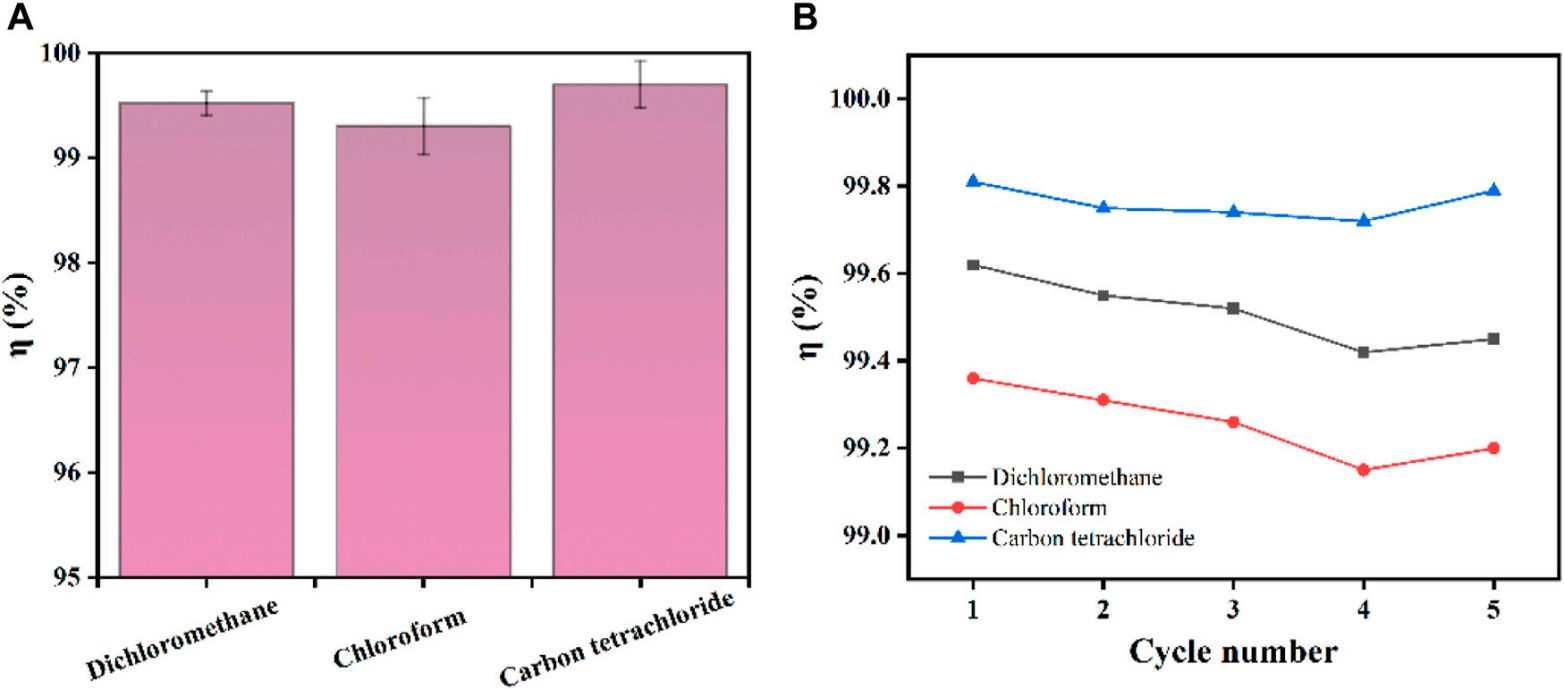
**(A)** Separation efficiency of superhydrophobic sponge for three heavy oils. **(B)** Separation efficiency of 5 cycles for separating three kinds of heavy oil.

Ag/SiO_2_@PDMS@CS modified sponges can also be used for heavy oil/water separation under gravity drive. The prepared superhydrophobic sponge is fixed between the oil and water separation devices. The heavy oil can permeate through the sponge, and the water is trapped above the device so that the heavy oil can be collected (Figure 10). As can be seen from Figure 11A, the sep-aration efficiency of super hydrophobic sponge for three heavy oils (methylene chloride, chlo-roform and carbon tetrachloride) is greater than 99.2%. After 5 cycles of oil-water separation experiments, the superhydrophobic sponge still showed high separation efficiency (*η*> 99%), after the fifth cycle, the WCA of the sponge remained above 155°, which verified the excellent stability of the sponge (Figure 11B). According to the above experimental results, superhydrophobic sponge has excellent oil absorption performance and oil-water separation ability, which can be attributed to its superhydrophobicity and high porosity.

### 3.4 Treatment of polymer-containing sewage

Oilfield polymer-containing wastewater samples mainly contain solid particles such as oil, polymer, and suspended matter, which are relatively stable. It is difficult to meet the requirements of wastewater treatment only through physical methods. This experiment combined the physical method and chemical method to treat the sewage samples and achieved good results. The treatment effects are shown in Table 1. As a flocculant, polyaluminum chloride damages the stability of the polymer, so that the oil beads and solid impurities in sewage float or settle by flocculation, and then use the superhydrophobic-superlipophilic of the superhydrophobic sponge for oil adsorption. Compared with the sewage sample, the oil content of the treated water is 10.40 mg/L, the polymer concentration is 2.8921 mg/L, the solid content of the suspended matter is about 21 mg/L, and the viscosity is reduced to 1.04 mPa s.

**TABLE 1:** Comparison of the treatment effect of polymer-containing wastewater before and after.

Sample	Oil content (mg/L)	Polymer concentration (mg/L)	Suspended solids content (mg/L)	Viscosity (mPa·s)	TOC (mg/L)
Before treatment	450	167.8	400.04	1.98	1.81×10^9^
After treatment	10.40	2.59	21	1.04	162.40

### 3.5 Antibacterial performance test

**FIGURE 12:**
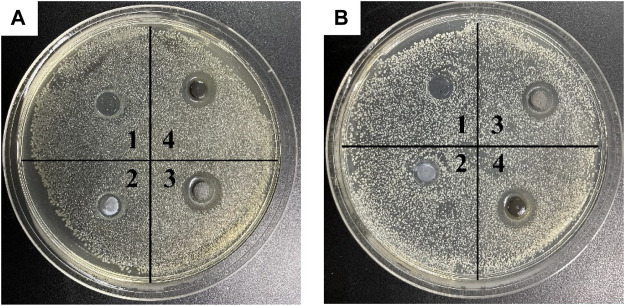
**(A)** Inhibition cycle of *E. coli* by acetone (1), SiO_2_ (2), Ag/SiO_2_ NPs (3) and CS@Ag/SiO_2_ NPs@PDMS. **(B)** Antibacterial cycle of acetone (1), SiO_2_ (2), Ag/SiO_2_ NPs (3) and CS@Ag/SiO_2_ NPs@ PDMS against *Staphylococcus aureus*.

The bacteriostatic zone method can quickly evaluate the ability of materials to inhibit bacterial reproduction. Numbers 1, 2, 3 and 4 in Figures 12A, B refer to acetone, SiO_2_ NPs, Ag/SiO_2_ NPs, CS@Ag/SiO_2_ NPs@PDMS, respectively.By comparison, no bacteriostatic zone was found in No. 1 and No. 2, indicating that acetone and SiO_2_ NPs had no antibacterial effect on the two bacteria.There were obvious bacteriostatic zones around No. 3 and No. 4, indicating that Ag/SiO_2_ NPs and CS@Ag/SiO_2_ NPs@PDMS had good antibacterial effects. In addition, it also indicated that the addition of CS and PDMS did not change their original bacteriostatic properties, but slightly in-creased their bacteriostatic properties. Ag/SiO_2_ NPs had inhibition zones of 12 ± 0.20 mm and 15 ± 0.12 mm for *Escherichia coli* and *Staphylococcus aureus*, respectively, while CS@Ag/SiO_2_ NPs@PDMS had inhibition zones of 13 ± 0.12 mm and 15 ± 0.15 mm for *Escherichia coli* and *Staphylococcus aureus*, respectively.

**FIGURE 13:**
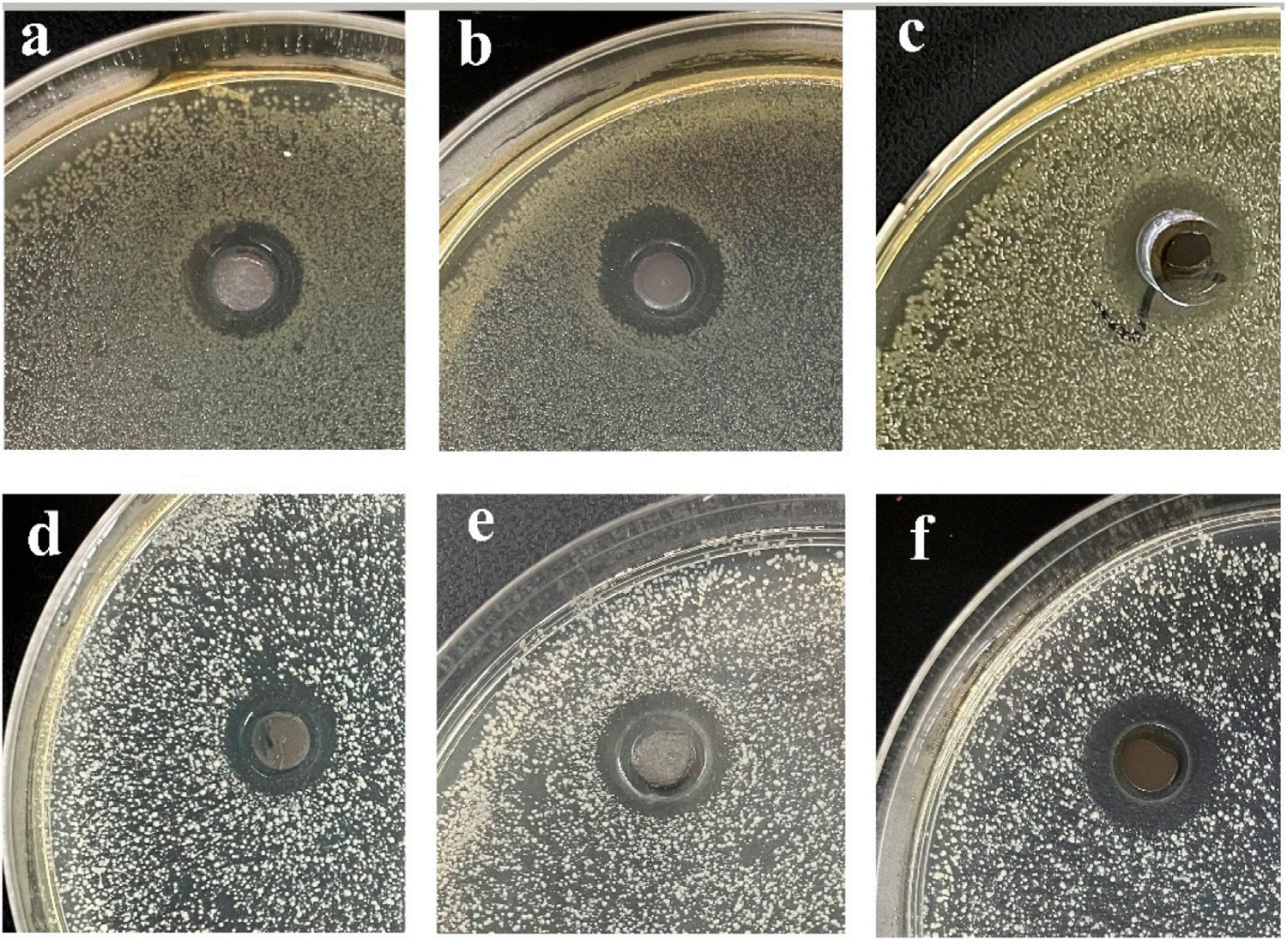
**(A-C)** Antibacterial cycle of Ag/SiO_2_ NPs @PDMS@CS with different Ag/SiO_2_ NPs contents against *E. coli*, **(D-F)** Bacteriostatic cycle of CS @Ag/SiO_2_ NPs @PDMS with different Ag/SiO_2_ NPs contents against *Staphylococcus aureus*.

Figure 13 Compared the antibacterial activities of CS@Ag/SiO_2_ NPs @PDMS with different Ag/SiO_2_ NPs contents against *Escherichia coli* (a-c) and *Staphylococcus aureus* (d-f). As can be seen from the figure, they all have a relatively obvious bacteriostatic zone. For the two bacteria, with the increase of Ag/SiO_2_ NPs content, the corresponding Ag NPs content increased, so the antibacterial zone also increased gradually. For *Escherichia coli*, the antibacterial zone diameter was 11 ± 0.08 mm, 13 ± 0.14 mm, 15 ± 0.11 mm, and for *Staphylococcus aureus*, the antibacterial zone diameter was 14 ± 0.05 mm, 15 ± 0.10 mm, 17 ± 0.12 mm, respectively.

## 4 Conclusion

We proposed a method for preparing super hydrophobic/super oleophilic sponges. Firstly, Ag/SiO_2_ NPs with antibacterial properties was prepared, with CS@Ag/SiO_2_ NPs@PDMS mixed solution as the super hydrophobic base, and then the sponge was modified by simple spraying method.The resulting superhydrophobic sponge has excel-lent hydrophobicity, oil/water separation and antibacterial properties.The hydrophobic CS particles decorated on the surface of the sponge improve the surface roughness, and the PDMS coating acts as a binding layer between the nanoparticles and the substrate, thus achieving a superhydrophobic surface.The superhydrophobic CS@Ag/SiO_2_ NPs@PDMS sponge has a separation efficiency of up to 99.8% for various oil-water mixtures, and can effectively purify polymer-containing wastewater in oilfield.At the same time, the super-hydrophobic CS@Ag/SiO_2_ NPs@PDMS coating also showed excellent antibacterial per-formance, with a bacteriostatic zone of 15 ± 0.15 mm for *Escherichia coli* and 17 ± 0.12 mm for *Staphylococcus aureus*. The prepared CS@Ag/SiO_2_ NPs@PDMS mixed solution can also be applied to different surfaces using spray technology to achieve a superhydro-phobic surface. The preparation method reported in this study is simple, green and low cost, which can provide a new idea for the development of multifunctional oil-water separation materials.

## Data Availability

The original contributions presented in the study are included in the article/supplementary material, further inquiries can be directed to the corresponding author.

## References

[B1] Ahuja D. Dhiman S. Rattan G. Monga S. Singhal S. Kaushik A. (2021). Superhydrophobic modification of cellulose sponge fabricated from discarded jute bags for oil water separation. J. Environ. Chem. Eng. 9 (2), 105063. 10.1016/j.jece.2021.105063

[B2] Baig U. Faizan M. Waheed A. (2022b). A review on super-wettable porous membranes and materials based on bio-polymeric chitosan for oil-water separation. Adv. Colloid Interface Sci. 303, 102635. 10.1016/j.cis.2022.102635 35325601

[B3] Baig U. Gondal M. A. Dastageer M. A. (2022a). Oil-water separation using surface engineered superhydrophobic and superoleophilic membrane for the production of clean water. J. Water Process. Eng. 45, 102473. 10.1016/j.jwpe.2021.102473

[B4] Barthwal S. Jeon Y. J. Lim S. H. (2022). Superhydrophobic sponge decorated with hydrophobic MOF-5 nanocoating for efficient oil-water separation and antibacterial applications. Sustain. Mater. Technol. 33, e00492. 10.1016/j.susmat.2022.e00492

[B5] Cao S. Li B. Zhu R. M. Pang H. (2019). Design and synthesis of covalent organic frameworks towards energy and environment fields. Chem. Eng. J. 355, 602–623. 10.1016/j.cej.2018.08.184

[B6] Farrokhi S. J. Pakzad H. Fakhri M. Moosavi A. (2021). Superhydrophobic home-made polyurethane sponges for versatile and cost-effective oil and water separation. Sep. Purif. Technol. 276, 119240. 10.1016/j.seppur.2021.119240

[B7] Kang L. Shi L. J. Song L. F. Guo X. P. (2022). Facile fabrication of superhydrophobic porous materials using the water-based aza-Michael reaction for high-efficiency oil-water separation. Sep. Purif. Technol. 308, 122880. 10.1016/j.seppur.2022.122880

[B8] Kong Y. Zhang S. M. Gao Y. Qi Y. F. Xiaohu C. Wang S. Q. (2022). Low-temperature carbonization synthesis of carbon-based super-hydrophobic foam for efficient multi-state oil/water separation. J. Hazard. Mater. 423, 127064. 10.1016/j.jhazmat.2021.127064 34537651

[B9] Li C. Y. Li J. P. Wang N. N. Zhao Q. Wang P. (2021). Status of the treatment of produced water containing polymer in oilfields: A review. J. Environ. Chem. Eng. 9, 105303. 10.1016/j.jece.2021.105303

[B10] Liu G. N. (2021). Experimental study on indoor filtration treatment of polymer-containing wastewater. Contemp. Chem. In-dustry 50, 316. 10.13840/j.cnki.cn21-1457/tq.2021.02.015

[B11] Pang Y. Yu Z. X. Chen H. D. Xiang Q. C. Wang Q. X. Xie C. X. (2022). Superhydrophobic polyurethane sponge based on sepiolite for efficient oil/water separation. J. Hazard. Mater. 434, 128833. 10.1016/j.jhazmat.2022.128833 35429755

[B12] Qiao S. Y. Liu Q. W. Fan Z. Z. Tong Q. L. Cai L. Fu Y. F. (2022). Magnetic hyperbranched molecular materials for treatment of oily sewage containing polymer in oilfield compound flooding. Front. Chem. 10, 865832. 10.3389/fchem.2022.865832 35665059 PMC9157815

[B13] Wang B. Z. Wang W. Zhang Y. R. Ma S. Yang X. Feng Y. Z. (2022). Superhydrophobic porous polyvinylidene fluoride monolith with outstanding environmental suitability for high-efficient continuous oil/water separation under harsh conditions. J. Environ. Chem. Eng. 10 (3), 107480. 10.1016/j.jece.2022.107480

[B14] Wang F. Chang R. R. Ma R. R. Tian Y. Q. (2021). Eco-friendly and superhydrophobic nano-starch based coatings for self-cleaning application and oil-water separation. Carbohydr. Polym. 118410, 118410. 10.1016/j.carbpol.2021.118410 34364553

[B15] Xu J. Q. Ren D. F. Chen N. Li X. F. Ye Z. X. Ma S. Y. (2021). A facile cooling strategy for the preparation of silica na-noparticles with rough surface utilizing a modified Stöber system. Colloids Surf. A Physicochem. Eng. Asp. 625, 126845. 10.1016/j.colsurfa.2021.126845

[B16] Yang S. D. Li H. Y. Liu S. Wang S. S. Li H. M. Li H. M. (2022). Wodyetia bifurcate structured carbon fabrics with durable superhydrophobicity for high-efficiency oil-water separation. J. Hazard. Mater. 439, 129688. 10.1016/j.jhazmat.2022.129688 36104914

[B17] Yu H. L. Wang F. Sun C. L. Liu H. S. Tang L. Wang Y. J. (2022). Evaluation of the self-assembled functional PPFS-P-AM composite for treating oilfield sewage. Sci. Total Environ. 833, 155228. 10.1016/j.scitotenv.2022.155228 35421506

[B18] Zhang L. Li H. Q. Lai X. J. Su X. J. Liang T. Zeng X. R. (2017). Thiolated graphene-based superhydrophobic sponges for oil-water separation. Chem. Eng. J. 316, 736–743. 10.1016/j.cej.2017.02.030

